# Association of HLA DRB variants to genetic predisposition to rheumatoid arthritis

**DOI:** 10.1186/1471-2164-15-S2-P32

**Published:** 2014-04-02

**Authors:** Bushra S Jarullah

**Affiliations:** 1 Department of Biotechnology, Kadi Sarva Vishwavidyalaya, Gandhinagar, Gujarat, India

## Background

Genetic predisposition to rheumatoid arthritis related to the presence of specific polymorphic HLA sequences has been frequently reported [1,2]. HLA class II is a heterodimer consisting of an alpha (DRA) and a beta (DRB) chain. Within the DR molecule the beta chain contains all the polymorphisms specifying the peptide binding specificities. Association studies have provided evidence that a sequence of amino acids, termed the shared epitope, are involved in the disease process [3].

## Materials and methods

In order to understand the influence of variation in HLA DRB genes on rheumatoid arthritis sequence comparison of 21 allelic variants of HLA DRB gene was carried out.

## Results

Although a lot of variation was observed in the 5’ ends, almost all sequences showed alignment scores of above 200 for 30-40% of the sequence towards the 3’ end. High levels of variations were however observed in two alleles; HLA-DRB1- 1318 and HLA-B*5904. Both these alleles have been recently reported as novel variants of HLA [4,5].

## Conclusions

Our results strongly support the shared epitope hypothesis presenting an explanation for the susceptibility to rheumatoid arthritis. Yet considering the length of similar sequence at the 3’ end and the hyper variable region at the 5’ end, we conclude the hypothesis may not completely explain the course of disease severity and emphasize the need to develop superior biomarkers to predict the course of the disease.
